# Anti-DHAV-1 reproduction and immuno-regulatory effects of a flavonoid prescription on duck virus hepatitis

**DOI:** 10.1080/13880209.2017.1309554

**Published:** 2017-04-07

**Authors:** Yun Chen, Ling Zeng, Jingjing Yang, Yixuan Wang, Fangke Yao, Yi Wu, Deyun Wang, Yuanliang Hu, Jiaguo Liu

**Affiliations:** Institute of Traditional Chinese Veterinary Medicine, College of Veterinary Medicine, Nanjing Agricultural University, Nanjing, P R China

**Keywords:** Duck hepatitis A virus type 1, baicalin-linarin-icariin-notoginsenoside R1, virus adsorption, virus replication, virus release

## Abstract

**Context:** The flavonoid prescription baicalin-linarin-icariin-notoginsenoside R1 (BLIN) has a curative effect on duck virus hepatitis (DVH) caused by duck hepatitis A virus type 1 (DHAV-1). However, the mechanism of this curative effect is not understood.

**Objective:** This study investigates the mechanism of the curative effect of BLIN on DVH caused by DHAV-1. We analyzed the anti-DHAV-1 reproduction mechanism and immuno-regulatory effect of BLIN.

**Materials and methods:** The anti-DHAV-1 reproduction effects of BLIN at 20, 10, 5 and 2.5 μg/mL *in vitro*, as well as the influence of BLIN at 20 μg/mL on DHAV-1 adsorption, replication and release were tested using the qRT-PCR method. The promotion abilities of BLIN at 20, 10, 5 and 2.5 μg/mL on T- and B-lymphocyte proliferation were investigated by the MTT method. IL-2 and IFN-γ levels and total anti-DHAV-1 antibody secretion after treatment with DHAV-1 for 4, 8 and 54 h were determined by ELISA.

**Results:** BLIN showed a dose-dependent DHAV-1 reproduction inhibitory effect. The inhibitory effect was highest at 20 μg/mL, where DHAV-1 adsorption and release were significantly lower. Meanwhile, BLIN at 5 μg/mL significantly increased T and B lymphocyte proliferation. BLIN stimulated total anti-DHAV-1 antibody secretion in ducklings at the dosage of 4 mg per duckling, but did not stimulate IL-2 and IFN-γ secretion significantly.

**Conclusions:** BLIN inhibits DHAV-1 reproduction by suppressing its adsorption and release. Additionally, BLIN promoted the duckling antiviral response.

## Introduction

Duck virus hepatitis (DVH) caused by duck hepatitis A virus (DHAV) is a serious, life-threatening disease in ducklings (Woolcock 2003). The DHAV genus is genetically divided into three types: DHAV type 1 (DHAV-1), DHAV type 2 and DHAV type 3 (Kim et al. 2009). Among these, DHAV-1 is common and widely distributed. To date, there is no effective antiviral drug cure. Hence, the development of an effective drug against DHAV-1 is urgently needed.

Recently, many investigators have focused on Chinese medicinal herb extraction to develop new antiviral drugs (Li et al. 2015; Moghaddam et al. 2015; Song et al. 2015). Flavonoids are important ingredients isolated from Chinese medicinal herbs. They have anti-inflammatory, antibacterial, antifungal, antiviral, antioxidative and immuno-enhancing activities (Orhan et al. 2010; Sithisarn et al. 2013; Fan et al. 2014; Panat et al. 2016). It is our hope to exploit Chinese medicinal herbs as a source of antiviral drugs such as an anti-DHAV-1 agent. In our previous study, the flavonoid prescription drug baicalin-linarin-icariin-notoginsenoside R1 (BLIN) showed a curative effect on DVH caused by DHAV-1, significantly reducing DHAV-1 reproduction in ducklings (Chen et al. 2017). However, its therapeutic mechanism is not clear.

Many antiviral drugs block one or more stages of the viral life cycle, including initial attachment to the target cell, viral replication and viral release (Harden et al. 2009; Song et al. 2013; Chen et al. 2014a). To understand in depth how BLIN reduces DHAV-1 reproduction, it is necessary to study whether BLIN blocks the life cycle of DHAV-1 and what stage(s) DHAV-1 is affected by BLIN. Additionally, the progress of the viral disease is closely related to the animal’s immune level (Pettersson et al. 2014), and the strength of immuno-competence often determines animal survival rates. Whether BLIN regulates the immune response when used to treat DVH is unknown.

To investigate the immuno-regulation effect and anti-DHAV-1 mechanism of BLIN, we designed several experiments. The anti-DHAV-1 reproduction ability of BLIN was determined and the anti-DHAV-1 reproduction mechanisms were detected by quantitative reverse transcriptase polymerase chain reaction (qRT-PCR). Additionally, the influence of BLIN on T and B lymphocyte proliferation was tested. Meanwhile, the stimulatory effect of BLIN on IL-2, IFN-γ and total anti-DHAV-1 antibody secretion was detected.

## Materials and methods

### Reagents and virus

The growth medium (GM) was Dulbecco’s modified eagle medium (Gibco, Grand Island, NY) supplemented with penicillin 100 IU/mL, streptomycin 100 μg/mL and 10% foetal bovine serum; in the maintenance medium (MM), the foetal bovine serum was 1%. RPMI-1640 (Gibco) was supplemented with penicillin 100 IU/mL, streptomycin 100 μg/mL and 10% foetal bovine serum. Trypsin (Amresco, Solon, OH) was dissolved into 0.2% with Dulbecco’s Hanks Balanced Salt Solution (D-Hank’s). Both phytohaemagglutinin (PHA, Sigma) and lipopolysaccharide (LPS, Sigma), as the T-cell mitogen and the B-cell mitogen, respectively, were dissolved at 100 μg/mL in RPMI-1640. 3-(4,5-Dimethylthiazol-2-yl)-2,5-diphenyltetrazolium bromide (MTT, Amresco) was dissolved in calcium- and magnesium-free phosphate-buffered saline at 5 mg/mL. Heparin sodium was dissolved at 2 mg/mL in physiological saline. The lymphocyte separation medium (G20150910-1059) was the product of the Tianjin Hao Yang Biological Manufacture Company, China. RNAiso Plus Reagent (Lot no. 9251), PrimeScript^TM^ RT Master Mix Kit (Lot no. AK3101) and SYBR^®^ Premix Ex Taq^TM^ (Tli RNaseH Plus) Kit (Lot no. AK5903) were purchased from Takara, Japan. DHAV (*LQ_2_* strain) was supplied by the Shandong Institute of Poultry in China.

### BLIN

BLIN was prepared in our laboratory. It was composed of four flavonoids: baicalin (98.00%), linarin (95.00%), icariin (98.00%) and notoginsenoside R1 (93.00%). All these flavonoids were purchased from Nanjing Zelang Medicine Science and Technology Co., Ltd. (Nanjing, Jiangsu Province, China). The ratio of baicalin: linarin: icariin: notoginsenoside R1 was 4:2:16:1 (Chen et al. 2017).

### Animals and cells

#### Animals and ethics statement

All the animals were purchased from the Tangquan Poultry Farm, Jiangsu province, China. All animal experiments in our work conformed to the Guide for the Care and Use of Laboratory Animals published by the US National Institutes of Health (NIH Publication, Eighth edition, 2011) and was approved by the Nanjing Agricultural University Animal Care Committee.

#### Duck embryonic hepatocytes (DEHs)

DEHs were prepared according to the method described previously (Chen et al. 2014a). First, the tissue was collected, minced and washed three times with D-Hank’s. Next, the tissue was digested with a solution of 0.20% trypsin. The tissue was washed three times with D-Hank’s after removing the redundant trypsin. The cells were then cultured in GM in a humid atmosphere of 5% CO_2_ at 37 °C. When the hepatocytes grew into a monolayer, the GM was removed and the cells were collected for standby.

#### B lymphocytes

Splenic B lymphocytes were prepared according to the method of Zhao et al. (2012). A spleen from a non-immunized 30-day-old cherry valley duck was gently ground and then diluted with D-Hank’s. The diluted spleen sample was carefully and slowly layered on the surface of the lymphocyte separation medium in a centrifuge tube. After 10 min of centrifugation at 1500 *g*, the B lymphocytes were collected and washed twice with D-Hank’s. Finally, the B lymphocytes were diluted to 2.5 × 10^6^ cells/mL with RPMI-1640 with foetal bovine serum and collected for standby.

#### T lymphocytes

Peripheral T lymphocytes were prepared according to the method of Fan et al. (2012). The blood sample was collected from a non-immunized 30-day-old cherry valley duck and immediately diluted by quadruple D-Hank’s containing sodium heparin. The diluted blood sample was carefully layered on the surface of the lymphocyte separation medium in a centrifuge tube. After centrifugation at 1000 *g* for 10 min, the T lymphocytes were collected and washed twice with D-Hank’s. Finally, the T lymphocytes were diluted to 2.5 × 10^6^ cells/mL with RPMI-1640 and collected for standby.

#### Anti-DHAV-1 reproduction effect assay

A 24-well cell culture plate containing a DEHs monolayer was treated with 200 μL DHAV-1 (100 TCID_50_) per well, except for the control wells. In the meantime, 200 μL BLIN at 40, 20, 10 and 5 μg/mL (the working concentrations were, respectively, 20, 10, 5 and 2.5 μg/mL) was added to the BLIN-treated wells. The cell control and virus control wells were made up to 400 μL with MM. Then, the DHAV-1 was diluted to 50 TCID_50_ and BLIN was diluted to 20 μg/mL (the concentration had no toxicity to DEHs as determined by a pre-experiment cytotoxicity test), 10, 5 and 2.5 μg/mL. The plate was incubated at 37 °C in a humid atmosphere of 5% CO_2_ for 24 h. Finally, the qRT-PCR method was applied to measure the DHAV-1 reproduction level.

### Assay of antiviral process of BLIN during DHAV-1 viral life

#### Adsorption assay

The adsorption assay consisted of two sample-adding modes: pre-adding drug and post-adding drug modes (Chen et al. 2014a). Briefly, in the pre-adding drug mode, the virus control wells and BLIN-treated wells in a 24-well cell culture plate containing a DEHs monolayer were incubated with 400 μL MM and 400 μL BLIN (20 μg/mL), respectively, at 4 °C for 4 h. Then, the plate was washed three times with D-Hank’s and 400 μL DHAV-1 (50 TCID_50_) was added to all wells. The plates were then incubated at 37 °C in a humid atmosphere of 5% CO_2_ for 1 h. After that, the qRT-PCR method was used to detect virus adsorption. In the post-adding drug mode, all wells of a 24-well cell culture plate containing a DEHs monolayer were incubated with 400 μL DHAV-1 (50 TCID_50_) at 37 °C in a humid atmosphere of 5% CO_2_ for 1 h. Then, the plate was washed three times with D-Hank’s and 400 μL MM (virus control wells) or 400 μL BLIN (BLIN-treated wells) was added. The plates were then incubated at 4 °C for 4 h. Similarly, the qRT-PCR method was used to detect virus adsorption. Cell controls were used in these two assays.

#### Replication assay

All wells of a 24-well cell culture plate containing a DEHs monolayer were treated with the addition of 400 μL DHAV-1 (50 TCID_50_). The plate was then incubated at 37 °C in a humid atmosphere of 5% CO_2_ for 2 h to allow adsorption and penetration (Song et al. 2013). After that, the plate was washed three times using D-Hank’s and 400 μL MM or 400 μL BLIN (20 μg/mL) were added to the virus control wells or the BLIN-treated wells, respectively. No treatment was added to the control wells. To avoid being affected by virus release and immediate re-adsorption, the qRT-PCR method was used to detect virus replication after the plate was incubated at 37 °C in a humid atmosphere of 5% CO_2_ for 10 h (Yao et al. 2016).

#### Release assay

The 24-well cell culture plate was treated with 400 μL DHAV-1, except for the control wells, and the plate was incubated at 37 °C in a humid atmosphere of 5% CO_2_ for 32 h to allow adsorption, penetration, replication and release (Yao et al. 2016).The plates were then washed three times with D-Hank’s and 400 μL MM was added to the virus and cell control wells and 400 μL BLIN (20 μg/mL) was added to the BLIN-treated wells. Then, the plate was incubated at 37 °C in a humid atmosphere of 5% CO_2_ for 1 h. The cell supernatant was centrifuged and the sediment (cellular debris and other impurities) was removed. Finally, 100 μL centrifuged supernatant and 100 μL DEH (1.0 × 10^6^ cells/mL) were mixed together. The qRT-PCR method was used to detect virus release.

#### qRT-PCR

The qRT-PCR in the anti-DHAV-1 mechanism assay was performed according to a method described previously (Chen et al. 2014a). In brief, total RNA was extracted using the RNAiso Plus Reagent. The reverse transcription assay was executed using the PrimeScript^TM^ RT Master Mix Kit and real-time PCR was performed using SYBR^®^ Premix Ex Taq^TM^ (Tli RNaseH Plus) Kit. The primers for DHAV-1 (forward, 5′-GCCACCCTTCCTGAGTTTGT-3′; reverse, 5′-TACCATTCCACTTCTCCTGCTT-3′), IL-2 (forward, 5′-CCAGGAACGGGATGCAATAT-3′; reverse, 5′-AAGCGGACAGCAAGTTAGGTAG-3′), IFN-γ (forward, 5′-AACGCAAGGCTGTGAGTGAG-3′; reverse, 5′-ACTGGCTCCTTTTCCTTTTGG-3′), and β-actin (forward, 5′-CTTTCTTGGGTATGGAGTCCTG-3′; reverse, 5′-TGATTTTCATCGTGCTGGGT-3′) were designed using Primer Premier Software (version 5.0).

### Lymphocyte proliferation assays

#### Splenic B lymphocyte proliferation assay

Four active concentrations of BLIN (40, 20, 10 and 5 μg/mL; the working concentrations were, respectively, 20, 10, 5 and 2.5 μg/mL) were used to stimulate the splenic B lymphocytes. These concentrations had no toxicity to B lymphocytes, as determined by a cytotoxicity test (not shown).

First, 80 μL B lymphocytes was added into each well of a 96-well cell culture plate. Then, the plate was divided into four parts: in B lymphocyte control wells, 120 μL RPMI-1640 was added; in the LPS wells, 20 μL LPS and 100 μL RPMI-1640 were added; in the BLIN-treated wells, 100 μL BLIN and 20 μL RPMI-1640 were added; and in the LPS-BLIN-treated wells, 20 μL LPS and 100 μL BLIN were added. The plate was incubated at 37 °C in a humid atmosphere of 5% CO_2_ for 48 h and cytoactivity was measured using the MTT colorimetric assay (Zhang et al. 2012). The B lymphocyte proliferation rate was calculated based on the formula: Proliferation rate (%) = (*A*_test group_−*Ā*_control group_)/*Ā*_control group_ × 100% (Chen et al. 2015b).

#### Peripheral T-lymphocyte proliferation assay

The design of the peripheral T-lymphocyte proliferation assay was similar to that of the splenic B-lymphocyte proliferation assay. The differences were that the LPS became PHA and the cells became T lymphocytes.

#### IL-2 and IFN-γ mRNA

T lymphocytes (1000 μL) were added into each well of a 6-well cell culture plate. Afterwards, 1000 μL BLIN at 10 μg/mL (the working concentration was 5 μg/mL which is the optimal stimulus concentration on T lymphocyte) was added into the BLIN-treated wells; 500 μL RPMI-1640 was added into the cell control wells; three wells per treating. Then, the plate was incubated at 37 °C in a humid atmosphere of 5% CO_2_ for 48 h and the qRT-PCR method was used to detect IL-2 and IFN-γ mRNA.

#### Influence of BLIN on IL-2, IFN-γ and total anti-DHAV-1antibody secretion

A total of 120 four-day-old ducklings were randomly divided into three groups: virus control, blank control and BLIN-treated groups. Every duckling in the virus control group and BLIN-treated group was intramuscularly injected with DHAV-1 (0.2 mL; 10 LD_50_). At the same time, the ducklings in a blank control group were intramuscularly injected with 0.2 mL physiological saline. Then, the ducklings of the BLIN-treated groups were treated with the aqueous solution at the dosage of 4 mg per duckling, once a day. After injecting DHAV-1 for 4, 8 and 54 h (Chen et al. 2015a, 2015b), blood samples were randomly taken from five ducklings per group. The serum was separated and the IL-2, IFN-γ and total anti-DHAV-1 antibody levels were determined by an ELISA kit (Kamiya Biomedical Company, Seattle, WA) (Chen et al. 2015b). All these kits were specific for ducks. The detection ranges of the IL-2 kit were from 15 to 500 ng/L; for the IFN-γ kit from 4 to 80 ng/L, and for the total anti-DHAV-1 antibody kit from 2 to 120 ng/L. The intra- and inter-assay coefficients of variation for the IL-2 kit were 9% and 11%, respectively; for the IFN-γ kit 9% and 11%, respectively; and for the total anti-DHAV-1 antibody kit 9% and 15%, respectively.

#### Statistical analysis

Statistical analyses were performed by Duncan’s multiple range tests or *t* tests using the SPSS Software Package v.20.0. Significant differences were considered as *p* < 0.05. In anti-DHAV-1 reproduction mechanism assays, the statistical analyses were performed by *t* tests using the SPSS Software Package v.20.0. All results were expressed as the mean ± standard deviation (SD).

## Results

### DHAV-1inhibitory effect of BLINon DEHs

**Figure 1.**
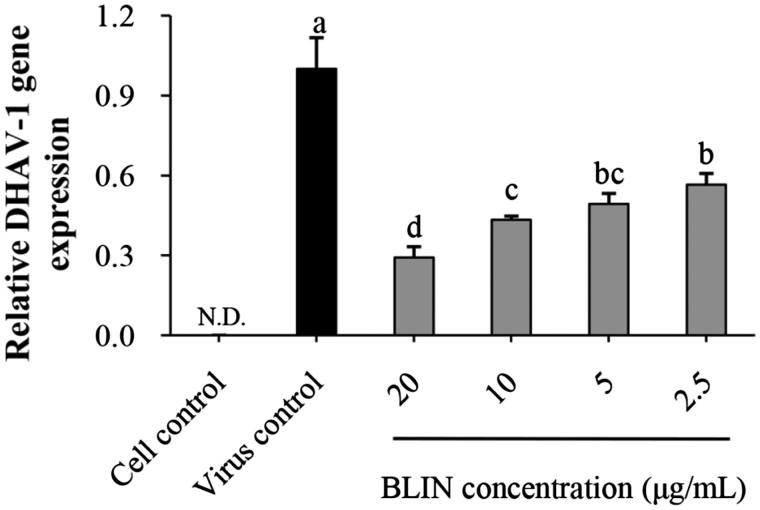
Anti-DHAV-1 reproduction effect of BLIN. When DEHs were infected with DHAV-1, BLIN at different working concentrations (20, 10, 5 and 2.5 μg/mL) was added to DEHs, five repetitions per treatment. After 24 h, the qRT-PCR method was used to measure the DHAV-1 reproduction level. Statistical analyses were performed using Duncan’s multiple range tests. ^a–d^Bars in the figure without the same superscripts differ significantly (*p* < 0.05). ND: not detected.

The anti-DHAV-1 reproduction effect of BLIN is depicted in Figure 1. BLIN at all test concentrations exhibited anti-DHAV-1 reproduction abilities. The relative DHAV-1 gene expression levels were significantly (*p* < 0.05) lower than that of the virus control. In addition, BLIN showed a dose-dependent DHAV-1 reproduction inhibitory effect. At 20 μg/mL, the DHAV-1 reproduction rate was the lowest, which was significantly (*p* < 0.05) lower than when the concentration was 10, 5 and 2.5 μg/mL.

### DHAV-1 reproduction inhibitory mechanism of BLIN on DEHs

**Figure 2.**
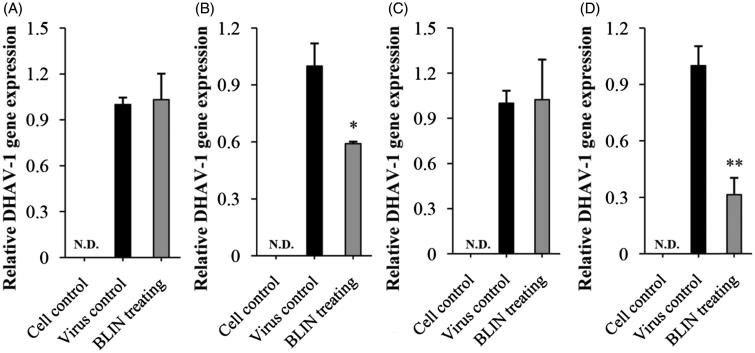
Influence of BLIN on DHAV-1 adsorption, replication and release. Two sample-adding modes, pre-adding drug (A) and post-adding drug (B) were used to observe the influence of BLIN on DHAV-1 adsorption. The assay in each mode was repeated five times. (C) To observe the influence of BLIN on DHAV-1 replication, DHAV-1 was added to DEHs for 2 h to allow adsorption and penetration of the virus, and then BLIN at 20 μg/mL was added to DEHs for 10 h, five repetitions per treatment. (D) To observe the influence of BLIN on DHAV-1 release, DHAV-1 was added to DEHs for 32 h to allow adsorption, penetration, replication and release, and then BLIN at 20 μg/mL was added to DEHs for 1 h, five repetitions per treatment. The qRT-PCR method was used to measure the relative DHAV-1 gene expression. Statistical analyses were performed by *t* tests. ND: not detected; *Compared with virus control, *p* < 0.05; **Compared with virus control, *p* < 0.01.

Figure 2 shows the influence of BLIN on DHAV-1 adsorption, replication and release. No gene expression was detected in the control cells, and the gene expression in the virus control in all assays was set to 1. For the adsorption assay, in the pre-adding drug mode (Figure 2(A)) there was no difference between the virus control and the BLIN-treated cells. In the post-adding drug mode (Figure 2(B)), BLIN significantly decreased (*p* < 0.05) the DHAV-1 content of DEHs. In the replication assay (Figure 2(C)), a difference between the virus control and the BLIN-treated cells was not apparent. For the release assay (Figure 2(D)), BLIN significantly reduced (*p* < 0.01) the detectable DHAV-1 quantity compared to the virus control.

### Lymphocyte proliferation effect of BLIN

#### Effect of BLIN on T-lymphocyte proliferation rate

**Figure 3.**
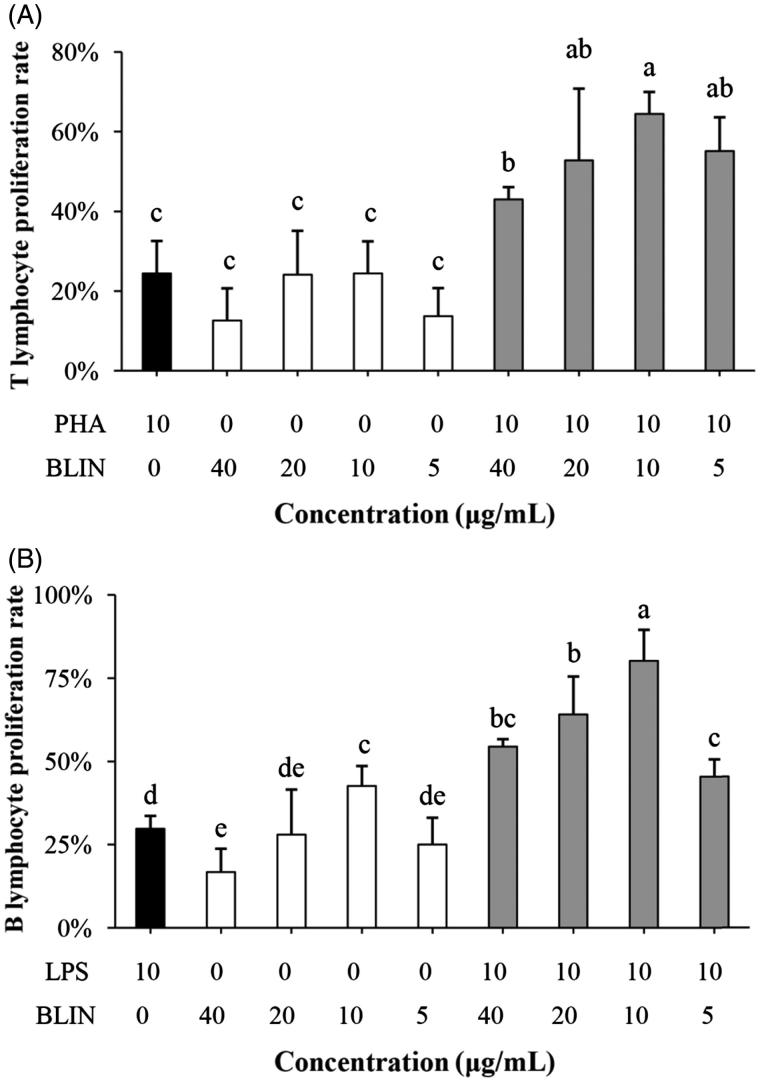
Influence of BLIN on lymphocyte proliferation. PHA, as a control was used to stimulate the T lymphocyte. (A) BLIN at different working concentrations (20, 10, 5 and 2.5 μg/mL) stimulated T lymphocytes singly or co-stimulated with PHA, five repetitions per treatment. LPS, as a control, was used to stimulate the B lymphocyte. (B) BLIN at different working concentrations (20, 10, 5 and 2.5 μg/mL) stimulated B lymphocytes singly or co-stimulated with LPS, five repetitions per treatment. Statistical analyses were performed using Duncan’s multiple range tests. ^a–e^Bars in the figure without the same superscripts differ significantly (*p* < 0.05).

The effect of BLIN on the peripheral T-lymphocyte proliferation rate is illustrated in Figure 3(A). The T-lymphocyte proliferation rate of PHA was 24.45%. BLIN stimulated peripheral T-lymphocyte proliferation at all test concentrations. The best concentration of BLIN for stimulating T-lymphocyte proliferation was 5 μg/mL, whether singly or co-stimulating with PHA. When BLIN stimulated T lymphocytes alone, the proliferation rate was 24.5%, which was similar to that of the PHA control. When BLIN co-stimulated T lymphocytes with PHA, the proliferation rate was 64.4%, which was significantly higher (*p* < 0.05) than that of the PHA control.

#### Effect of BLIN on B lymphocyte proliferation rate

The effect of BLIN on the splenic B-lymphocyte proliferation rate is illustrated in Figure 3(B). The results show that the B-lymphocyte proliferation rate of LPS is 29.73%. BLIN was able to stimulate splenic B lymphocyte proliferation effectively. At any test concentration, BLIN exhibited a stimulatory effect. The best concentration was 5 μg/mL, whether alone or co-stimulating with LPS. Whether BLIN was used alone or with LPS, the B-lymphocyte proliferation rates were significantly higher (*p* < 0.05) than that of the LPS control.

#### Influence of BLIN on the expression of IL-2 and IFN-γ mRNA

**Figure 4.**
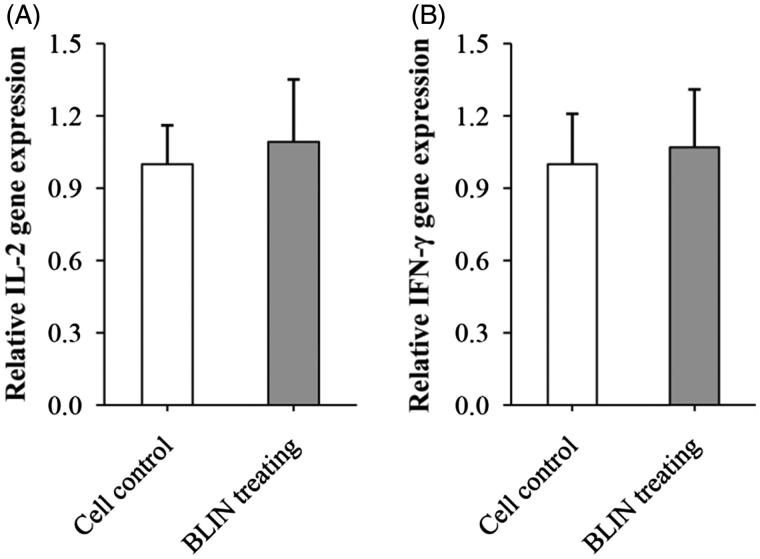
Influence of BLIN on the expression of IL-2 and IFN-γ mRNA. BLIN at 5 μg/mL was used to treat the B lymphocyte for 48 h. Then, the expression of IL-2 and IFN-γ mRNA was detected by qRT-PCR.

Figure 4 shows the influence of BLIN on the expression of IL-2 and IFN-γ mRNA. The relative IL-2 (Figure 4(A)) gene expressions of cell control and BLIN-treated group were at the same level. BLIN did not stimulate the IL-2 mRNA expression. Similarly, there was no significant difference between the IFN-γ (Figure 4(B)) gene expression of cell control and BLIN-treated group. So, BLIN did not stimulate the IFN-γ mRNA expression.

### Influence of BLIN on IL-2, IFN-γ and total anti-DHAV-1 antibody secretion

#### IL-2 secretion

**Figure 5.**
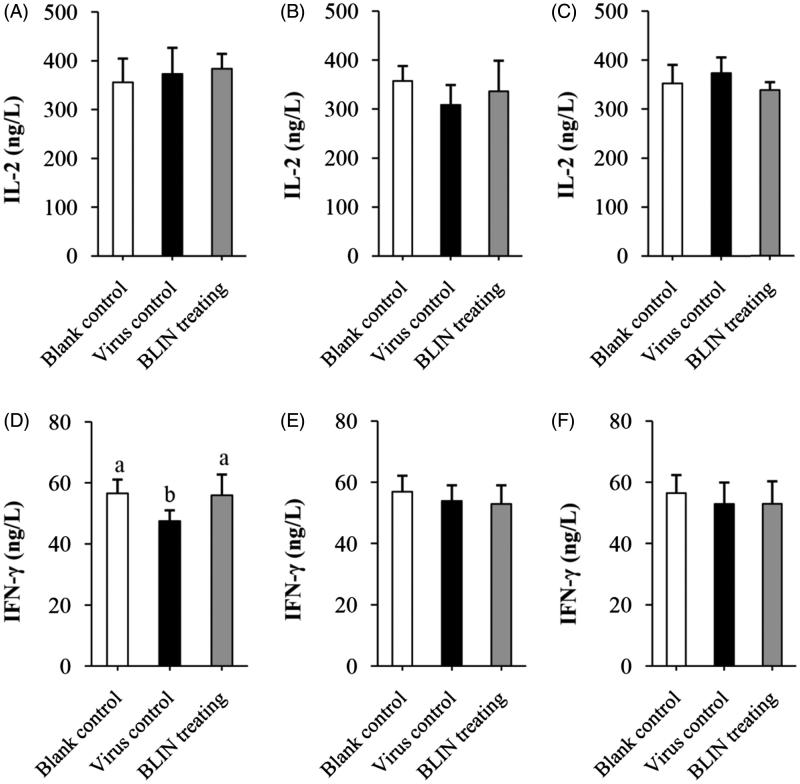
Influence of BLIN on IL-2 and IFN-γ secretion. After treatment with DHAV-1 for (A,D) 4, (B,E) 8 or (C,F) 54 h, the blood of ducklings in the blank control, virus control and BLIN treatment groups (five samples per group) was collected. The serum was separated, then IL-2 (A–C) and IFN-γ (D–F) levels were determined by an ELISA kit. Statistical analyses were performed using Duncan’s multiple range tests.

Figure 5 shows the influence of BLIN on IL-2 secretion. At 4 h (Figure 5(A)), 8 h (Figure 5(B)) and 54 h (Figure 5(C)) in the virus control ducklings and the BLIN-treated ducklings, the IL-2 content was always similar to that in the blank control ducklings. There was no significant difference between the virus control and BLIN-treated ducklings.

#### IFN-γ secretion

Figure 5 shows the influence of BLIN on IFN-γ secretion. At 4 h (Figure 5(D)) in the virus control duckling, the IFN-γ content significantly decreased (*p* < 0.05) compared to the blank control and BLIN-treated ducklings. There was no significant difference between the blank control and BLIN-treated ducklings. At 8 h (Figure 5(E)) and 54 h (Figure 5(F)), there was no significant difference in the IFN-γ content of the three groups.

#### Total anti-DHAV-1 antibody

**Figure 6.**
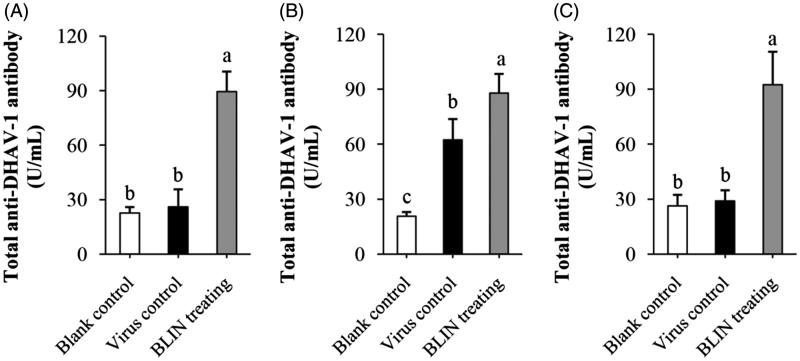
Influence of BLIN on total anti-DHAV-1 antibody secretion. After injecting DHAV-1 for (A) 4, (B) 8 or (C) 54 h, the blood of ducklings in the blank control, virus control and BLIN treatment groups (five samples per group) was collected. The serum was separated and total anti-DHAV-1 antibody levels were determined by an ELISA kit. Statistical analyses were performed using Duncan’s multiple range tests. ^a–c^Bars in the figure without the same superscripts differ significantly (*p* < 0.05).

Figure 6 shows the influence of BLIN on total anti-DHAV-1 antibody secretion. At 4 h (Figure 6(A)), the difference between the blank control and virus control had no statistical significance, while the total anti-DHAV-1 antibody level in the BLIN-treated ducklings was significantly higher (*p* < 0.05). At 8 h (Figure 6(B)), the total anti-DHAV-1 antibody level in the virus control ducklings was higher. It was significantly higher (*p* < 0.05) than in the blank control ducklings but was still significantly lower (*p* < 0.05) than in the BLIN-treated ducklings. At 54 h (Figure 6(C)), the total anti-DHAV-1 antibody level in the virus control ducklings was equivalent to the blank control level. The level in the BLIN-treated ducklings was still high and significantly higher than these two groups (*p* < 0.05).

## Discussion

DVH has spread worldwide since the first recognized outbreak in 1949 in Long Island, New York (Levine & Fabricant 1950). To ducklings, DHAV-1 is a highly pathogenic virus. When a duckling younger than three-weeks is infected with DHAV-1, death is inevitable (Woolcock 2003; Tseng et al. 2007). In China, vaccinating with the attenuated vaccine is scarce at many duck farms, especially those supplying food. In addition, outbreaks of DVH still occur in many ducklings that have been vaccinated with attenuated vaccine (Li et al. 2013). Even worse, no effective drug is able to cure this severe disease. In our previous study, we found that the flavonoid prescription BLIN exhibited a curative effect on DVH (Chen et al. 2017). To understand how BLIN cured DVH caused by DHAV-1, we explored the influence of BLIN on DHAV-1 reproduction and its immuno-regulation ability in this study.

In our previous study, BLIN was able to reduce the DHAV-1 content in ducklings infected with DHAV-1 (Chen et al. 2017). However, it was not known whether it directly acted on DHAV-1. In this paper, we studied the direct effect of BLIN on DHAV-1 reproduction in DEHs. After DHAV-1 infection of DEHs for 24 h, it had completed several reproduction cycles (Yao et al. 2016). Thus, the DHAV-1 amount in the DEHs was large. After treatment with BLIN, the DHAV-1 amount was significantly lower in comparison to the virus control. The results indicated that BLIN reduced the DHAV-1 content directly; thus, BLIN was able to inhibit DHAV-1 reproduction of DEHs. In addition, the inhibitory effect was highest when the concentration of BLIN was 20 μg/mL. Therefore, 20 μg/mL was selected as the active concentration of BLIN for the experiments *in vitro*.

Viruses infect the host cell and reproduce in the cell. The viral life cycle includes attachment to the target cell, replication (including virus assembly) in the target cell and release from the target cell. Many antiviral drugs work by blocking one or more of these steps (Liu & Thorp 2002; Damonte et al. 2004; Chen et al. 2014a). The influence of BLIN on the viral life cycle of DHAV-1 was detected in this study and was assayed using qRT-PCR. This method has been widely applied to the diagnosis and detection of DHAV-1 (Luo et al. 2008; Chen et al. 2014a; Yao et al. 2016). Because of its high specificity, high sensitivity and high automation, it was used in the present study to detect DHAV-1 at all stages of its viral life cycle.

To a virus, binding to the cell surface receptor is a critical step in cell invasion (Song et al. 2013; Damonte et al. 2004). Some Chinese medicinal herb extractions, such as *Radix Chuanmingshinis Violacei* polysaccharide (Song et al. 2013), interfere with the initial adsorption of the virus. A similar interference activity for BLIN was found in the present study. Interestingly, BLIN inhibited virus adsorption only in the post-adding drug mode. In the pre-adding drug mode, BLIN did not have an inhibitory effect on DHAV-1 adsorption. That might be because BLIN destroyed the binding between DHAV-1 and DHEs but did not enhance the resistibility of DEHs against DHAV-1 binding. The inhibitory ability of BLIN against DHAV-1 adsorption has important implications for host cells against infection. *Picorna* viruses undergo rapid replication and translation in cells to escape the defence mechanism of host cells (Andersson et al. 2005). Therefore, the inhibition of DHAV-1 replication is a benefit to the antiviral treatment. Many Chinese medicinal herb extractions have anti-DHAV-1 ability because of their inhibitory effects against DHAV-1 replication. Bush Sophora Root polysaccharide and icariin have anti-DHAV-1 ability both *in vitro* and *in vivo*, and their DHAV-1 replication inhibition ratios are 55.8% (Chen et al. 2014a) and 45.1% (Xiong et al. 2015), respectively. However, in the present study, BLIN did not reduce DHAV-1 replication in DEHs (Figure 2(C)), but it did exhibit a strong inhibition of DHAV-1 release from DHEs. Beginning at 32 h, DHAV-1 release reached a steady level (Yao et al. 2016). Therefore, the cellular supernatant at 33 h after treatment with or without BLIN for 1 h was collected and the DHAV-1 gene was detected by the qRT-PCR method. After treatment, BLIN inhibited 68.6% of DHAV-1 release. The low level of DHAV-1 release causes a lower reinfection rate for DEHs, which was very important to the survival of DEHs. These results implied that BLIN suppressed DHAV-1 reproduction by inhibiting its adsorption and release.

Our results indicated that BLIN inhibited DHAV-1 by a direct route. However, the *in vivo* environment is relatively complicated. Therefore, we asked whether there was an indirect action. We studied the immune-regulation ability of BLIN. Immuno-regulation is an important pathway for individual resistance to viruses (Fan et al. 2012; Chen et al. 2015b; Dabaghian et al. 2015; Isorce et al. 2015). For example, it is used successfully to cure hepatitis (Chen et al. 2015b; Isorce et al. 2015). The enhancing methods include stimulating lymphocyte proliferation and stimulating immunity-associated cytokine secretion (Fan et al. 2012; Chen et al. 2015b). T cells secrete IL-2, IFN-γ and other cytokines to improve cell-mediated immunity (Zhang et al. 2014; Batorov et al. 2015). B cells secrete antibodies to neutralize viruses (Bachmann et al. 1997). Flavonoids are able to enhance the immune response (Kang et al. 2006; Fan et al. 2012). Therefore, we tested the immune response in infected ducklings to verify whether the anti-DHAV-1 effect of BLIN was based on its immuno-regulation ability.

The T- and B-lymphocyte proliferation abilities of BLIN were tested. BLIN stimulated both T- and B-lymphocyte proliferation. BLIN at 10 μg/mL had the largest stimulatory effect. PHA and LPS are a T-cell mitogen and a B-cell mitogen, respectively, and were able to stimulate T- and B-lymphocyte proliferation (Chen et al. 2015b). In the present study, the T lymphocyte-stimulating ability of BLIN was the same as PHA and the B lymphocyte-stimulating ability of BLIN was significantly higher (*p* < 0.05) than LPS. These results suggested a strong growth promoting effect of BLIN on both T and B lymphocytes, especially on B lymphocytes. IL-2 and IFN-γ were secreted by T (Th1) cells, and they were two important cytokines representing the ability of the immune system. Many Chinese medicinal herb extractions could stimulate their secretion (Fan et al. 2012; Chen et al. 2013, 2015b). However, we found that BLIN did not increase the IL-2 and IFN-γ mRNA levels on T lymphocyte. And, it did not stimulate IL-2 secretion in infected ducklings and did not increase IFN-γ secretion, except at 4 h. This increase might be due to the DHAV-1 inhibition ability of BLIN because the IFN-γ content in virus control ducklings decreased but that in BLIN-treated ducklings was similar to the blank control. BLIN stimulated T lymphocyte proliferation but did not increase the IL-2 and IFN-γ contents, which indicated that BLIN did not stimulate the T helper cells, or at least not the Th1 subset, but other kinds of T cells. It also might be that the stimulating Th1 effect of BLIN did not work as fast as its DHAV-1 inhibition ability. However, the course of DVH is rapid and all infected ducklings died before 120 h of infection (Chen et al. 2014b, 2015a). Therefore, only if it works rapidly could BLIN treatment infect ducklings. Fortunately, BLIN stimulated total anti-DHAV-1 antibody secretion very quickly. After 4 h, the antibody was three times higher compared to the blank control ducklings. However, the total Abs generated at 4 and 8 h were probably primarily IgM (Chen et al. 2015b). We found that the increasing antibody level lasted a long time. At 54 h, the antibody level was still high, and the total Ab generated at this time point was probably primarily IgG (Chen et al. 2015b). When infected with virus, the antiviral immune response triggers (Battegay et al. 1996). At 8 h, the antibody in virus control ducklings also increased, but it was still significantly lower (*p* < 0.05) than in BLIN-treated ducklings. These results indicated that BLIN exhibited immuno-regulation ability and increased the duckling antiviral response.

In conclusion, BLIN has anti-DHAV-1 and immuno-regulation abilities. It inhibited DHAV-1 by suppressing DHAV-1 adsorption and release, and strongly promoted total anti-DHAV-1 antibody secretion, which resulted in its curative effect on DVH. It is worth studying the anti-DHAV-1 mechanisms of BLIN further to seek new therapeutic options for DVH.

## Conclusions

The immuno-regulation effect and anti-DHAV-1 reproduction mechanism of the flavonoid prescription BLIN were studied. BLIN has an anti-DHAV-1 ability and T and B lymphocyte-promoting effects. It also inhibited DHAV-1 reproduction by suppressing its adsorption and release. Additionally, BLIN stimulated total anti-DHAV-1 antibody secretion. BLIN has an immuno-regulation effect and inhibition ability against DHAV-1 adsorption and release.
